# Multi-Step Biomass Fractionation of Grape Seeds from Pomace, a Zero-Waste Approach

**DOI:** 10.3390/plants11212831

**Published:** 2022-10-25

**Authors:** Yara Salem, Hiba N. Rajha, Lambertus A. M. van den Broek, Carl Safi, Arnoud Togtema, Maria Manconi, Maria Letizia Manca, Espérance Debs, Zeina Hobaika, Richard G. Maroun, Nicolas Louka

**Affiliations:** 1 Centre d’Analyses et de Recherche, Unité de Recherche Technologies et Valorisation Agro-Alimentaire, Faculté des Sciences, Université Saint-Joseph de Beyrouth, Riad El Solh, P.O. Box 17-5208, Beirut 1104 2020, Lebanon; 2 Ecole Supérieure d’Ingénieurs de Beyrouth (ESIB), Université Saint-Joseph de Beyrouth, CST Mkalles Mar Roukos, Riad El Solh, Beirut 1107 2050, Lebanon; 3 Wageningen Food & Biobased Research, P.O. Box 17, 6700 AA Wageningen, The Netherlands; 4 Centre for Nanobiotechnology Sardinia CNBS, Department of Scienze della Vita e dell’Ambiente, University of Cagliari, 09124 Cagliari, Italy; 5 Department of Biology, Faculty of Arts and Sciences, University of Balamand, P.O. Box 100, Tripoli 1300, Lebanon

**Keywords:** natural extracts, grape byproducts valorization, biorefinery

## Abstract

Grape seeds are the wineries’ main by-products, and their disposal causes ecological and environmental problems. In this study seeds from the pomace waste of autochthonous grape varieties from Lebanon, Obeidi (white variety) and Asswad Karech (red variety) were used for a multi-step biomass fractionation. For the first step, a lipid extraction was performed, and the obtained yield was 12.33% (*w*/*w*) for Obeidi and 13.04% (*w*/*w*) for Asswad Karech. For the second step, polyphenols’ recovery from the defatted seeds was carried out, resulting in 12.0% (*w*/*w*) for Obeidi and 6.6% (*w*/*w*) for Asswad Karech, with Obeidi’s extract having the highest total phenolic content (333.1 ± 1.6 mg GAE/g dry matter) and antioxidant activity (662.17 ± 0.01 µg/mL of Trolox equivalent). In the third step, the defatted and dephenolized seeds were subsequently extracted under alkaline conditions and the proteins were isoelectric precipitated. The recovered protein extract was 3.90% (*w*/*w*) for Obeidi and 4.11% (*w*/*w*) for Asswad Karech seeds, with Asswad Karech’s extract having the highest protein content (64 ± 0.2 mg protein/g dry matter). The remaining exhausted residue can be valorized in cosmetic scrubs formulations as a replacement for plastic microbeads. The designed zero-waste approach multi-step biomass fractionation has the potential to improve the valorization of the side products (grape seeds) of these two Lebanese autochthonous grape varieties.

## 1. Introduction

Growing amounts of waste generated by the development of agro-industrial activities over the previous hundred years remain one of the key environmental challenges. If we consider the winemaking process, each stage produces its typical residues [1], including organic wastes (grape pomace containing seeds, pulp and skins, stems, and leaves), wine lees, and inorganic wastes [1,2]. Unfortunately, only a small percentage of the waste which remains is employed as fertilizers, animal feed, or other applications. Although winemaking is regarded as an eco-friendly process, it generates between 1.3 to 1.5 kg of waste per liter of wine produced, with 75% wastewater [3]. Moreover, a considerable portion of exhausted grape pomace is disposed in ponds and landfills, posing an environmental hazard mainly by attracting flies. The percolates produced by them deplete the oxygen in the soil and infiltrate surface, soil, and ground waters [4]. To minimize these effects, wine by-products can be valorized through the recovery of functional components or bioactive phytochemicals that can serve as pharmaceutical, food, and cosmetic ingredients [5]. Grape seeds are an abundant source of extractable phenolic compounds, such as benzoic acid (gallic acid) and cinnamic acid (coumaric acid, caffeic acid, and ferulic acid) next to flavonoids (catechin and epicatechin), anthocyanins, and proanthocyanins [6]. These natural molecules exhibit many health benefits, including antimutagenic and anticarcinogenic activities, antioxidant and anti-inflammatory effects, as well as the prevention and delay of cardiovascular diseases [7,8]. Furthermore, grape seeds contain carbohydrates, with glucose being the most abundant carbohydrate moiety [9]. Additionally, grape seed oil is known for its high amount of unsaturated fatty acids (90%), especially linoleic acid (58–78%) and oleic acid (15–20%) [10], and saturated fatty acids which include palmitic acid (7–10%) and stearic acid (4–6%) [11]. Grape seed oil, once extracted, includes natural antioxidants, fragrance, and color compounds [12]. Moreover, grape seeds contain proteins, with an amino acid composition that can vary depending on the grape variety, location, and fertilization conditions. This fraction can be valorized by enhancing the nutritional and sensory quality of food and winery products [13].

Due to the abundant presence of bioactive compounds in grape seeds, its fractioning and characterization remains a beneficial and valuable step for the valorization of these byproducts for application in the food, cosmetic, and nutraceutical industries. In this study two autochthonous Lebanese grape seeds varieties, Obeidi and Asswad Karech, were studied for the first time. Obeidi, a very rare white variety of Lebanese grape, is grown in the Beqaa Valley (Lebanon) and was categorized, by Chateau Saint-Thomas, as purely Lebanese. Asswad Karech, another red Lebanese native variety, also grown in the Beqaa Valley of Lebanon, is particularly known for its black skin berry.

The aim of this study was to identify, for the first time, the composition of seeds obtained from the pomace of these two Lebanese varieties, Obeidi (white) and Asswad Karech (red), and to fractionate the biomass in order to produce: first, lipids for food or energetic potential applications; second, polyphenols for cosmetic and food applications; third, protein for potential food supplementation; and fourth, an exhausted grape seed residue to substitute the plastic beads in scrubs. The approach is to set up a zero-waste eco-friendly process to valorize grape seeds.

## 2. Results and Discussion

### 2.1. Chemical Composition

**Figure 1:**
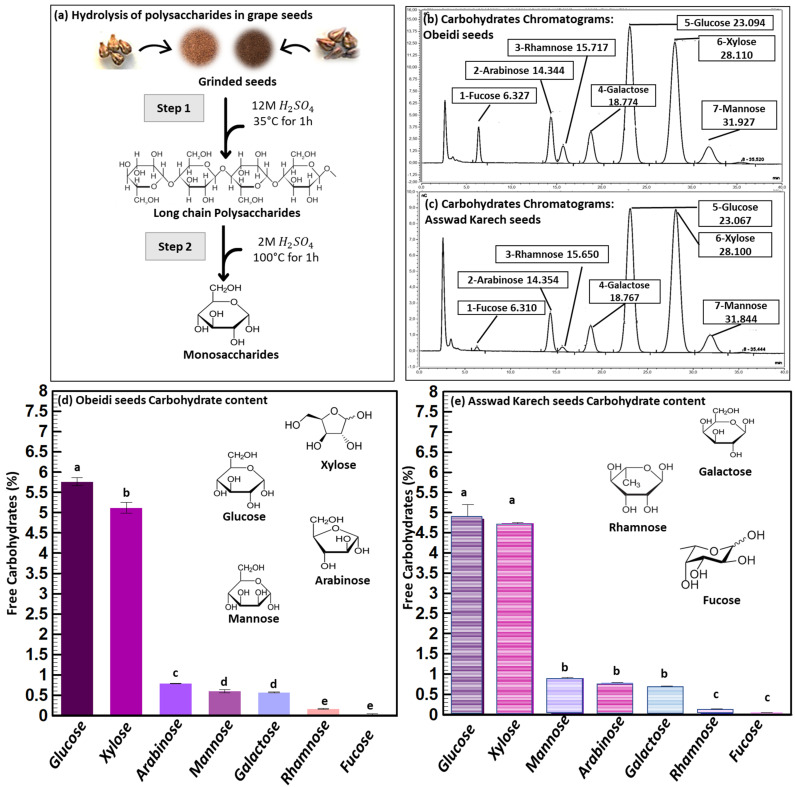
Carbohydrate analysis. (**a**) Schematic overview of the acid hydrolysis of carbohydrates, (**b**) chromatograms of hydrolyzed carbohydrates from Obeidi seeds, (**c**) chromatograms of hydrolyzed carbohydrates from Asswad Karech seeds, and (**d**,**e**) identification and quantification of carbohydrates in Obeidi and Asswad Karech grape seeds, respectively. Means sharing the same letter are not significantly different from each other (with *p >* 0.05).

The chemical composition of Obeidi and Asswad Karech grape seeds from pomaces was determined measuring the dry matter, ash content (Table 1) and carbohydrate composition (Figure 1). The two varieties had the same dry matter and ash content, without a significant difference.

The carbohydrate analysis of both grape seed samples was performed by converting the polymers into their carbohydrate moieties by sulfuric acid hydrolysis and the analysis was performed by High Performance Anion Exchange Chromatography (HPAEC) shown in Figure 1a. The most abundant carbohydrate was glucose (Figure 1b,c), which can originate from cellulose and/or starch. Xylose is a building block of arabinoxylan, and arabinose can be attached to the C2 and/or C3-position of xylose. Galactose and mannose are the building blocks of galactomannan. Rhamnose is a part of pectin and fucose can be attached to arabinoxylan. The total amount of carbohydrates (in their polymer form) was 13.05 ± 1% (*w*/*w*) of the dry matter content for Obeidi and 12.18 ± 1% (*w*/*w*) of the dry matter content for Asswad Karech. The comparison of the carbohydrate compositions underlined that both varieties have more or less a similar carbohydrate composition with minor differences. Valiente et al. found a higher amount of carbohydrates in the grape pomace of the variety Airén, with 22.7% (*w*/*w*) [14]. Here, glucose was the most abundant carbohydrate moiety after acid hydrolysis. However, the relative amount of arabinoxylan was much lower as found for the grape seeds without skins in our study.

For the concentrations of carbohydrates in Obeidi seeds, glucose was the most abundant one (5.76 ± 0.10%), followed by xylose (5.12 ± 0.13%), with a significant difference (*p* < 0.05). Lower concentrations were observed for arabinose (0.79 ± 0.01%), mannose and galactose (~0.59%; with no significant difference *p* > 0.05), and rhamnose and fucose (~0.10%; with no significant difference *p* > 0.05). Based on the results, seeds from the autochthonous Obeidi grape pomace (Figure 1d) are in agreement with the literature. Glucose is the most abundant monosaccharide found in the white grape seeds, followed in decreasing order by xylose, arabinose, mannose, and galactose, with the exception of fucose having a higher concentration than rhamnose, as reported by Valiente et al. for the Airén variety [14].

According to the concentrations of carbohydrates found in Asswad Karech, glucose was also the most abundant one (4.85 ± 0.35%). The other carbohydrate moieties showed the following concentrations, 4.74 ± 0.02% for xylose (with no significant difference with glucose *p* > 0.05), ~0.8% for mannose, arabinose, and galactose (with no significant difference; *p* > 0.05), and ~0.10% for rhamnose and fucose (with no significant difference; *p* > 0.05). However, a significance difference was found when comparing those three groups of carbohydrates to each other (*p* < 0.05).

Overall, the seeds from the pomace of Obeidi, the white grape variety, had a higher total amount of carbohydrates compared to Asswad Karech, the red grape variety. According to the literature, Harris et al. also reported a higher carbohydrate content in the seeds of white grape (Semillon) compared to that of the red variety (Shiraz), with glucose and xylose being the most abundant carbohydrates determined after acid hydrolysis [15].

Moreover, when comparing the composition of the two grape seeds varieties, the concentration of glucose and xylose was significantly higher in Obeidi than Asswad Karech (*p* < 0.05), whereas arabinose and rhamnose showed no significant differences (*p* > 0.05). On the other hand, the concentrations of mannose and galactose were significantly higher in seeds from Asswad Karech (*p* < 0.05). This indicates that Obeidi seeds have a higher starch and/or cellulose content, a less branched arabinoxylan, and a lower amount of galactomannan than Asswad Karech’s seeds. Due to the used acid hydrolysis condition, it was not possible to determine if the glucose originates from starch or cellulose.

### 2.2. Multi-Step Biomass Fractionation Grape Seeds

#### 2.2.1. First-Step: Lipid Recovery

Oil recovery is one of the most important routes for the valorization of grape pomace seeds, mainly due to the numerous valuable applications of seed oil in cosmetics, food supplements, and medicine [16]. Indeed, it is rich in bioactive components, which are suitable for use as a dietary supplement, to prevent and improve the physiological disorders of chronic diseases [16], or as cosmetic components. The oil recovery percentages are highly linked to the grape variety and were shown to range from 6 to 20% (*w*/*w*) when extracted via the Soxhlet method [17].

**Figure 2:**
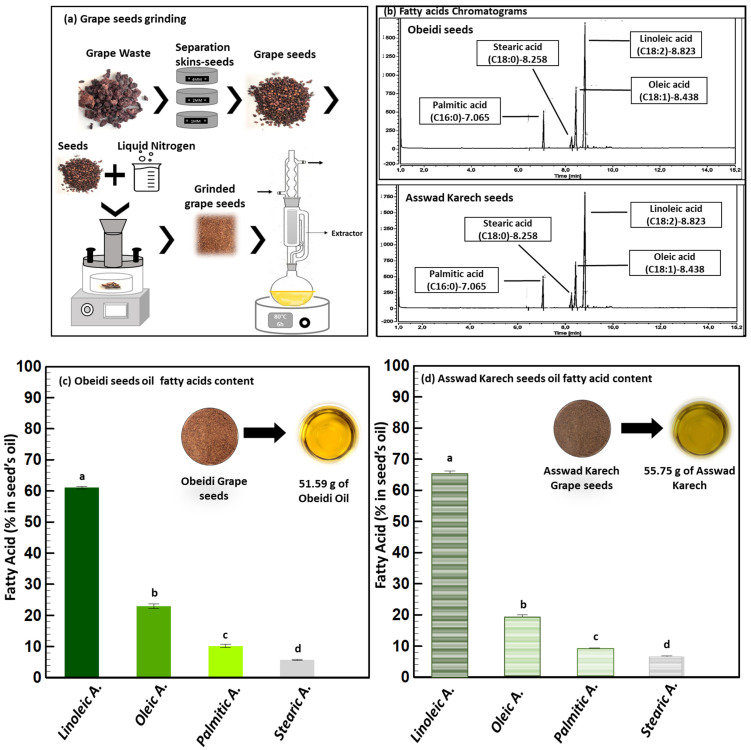
Lipid extraction. (**a**) Pretreatment (grinding) and extraction process, (**b**) GC chromatograms of Obeidi and Asswad Karech, and (**c**,**d)** identification and quantification fatty acids in Obeidi and Asswad Karech grape seeds, respectively. Means sharing the same letter are not significantly different from each other (with *p >* 0.05).

In the present study, before the first extraction step, the Obeidi and Asswad Karech seeds were separated from the pomaces, then were frozen and grinded (Figure 2a). The lipid extraction was conducted using a Soxhlet, and cyclohexane was used as the solvent (Figure 2a). Using this procedure, 12.33 ±0.1% (*w*/*w*) of yellow oil from the Obeidi seeds was recovered and 13.04 ± 0.1% (*w*/*w*) of greenish oil from the Asswad Karech (Equation (1)) was recovered. This is in agreement with the literature, as both autochthonous Lebanese varieties showed a recovery within the range of 10–20% [18]. The identification of the fatty acids in both oils was done by a Gas Chromatography-Flame Ionization Detector (GC-FID) (Figure 2b). The fatty acid content in the Obeidi grape seed oil is shown in Figure 2c. Linoleic acid was the most abundant fatty acid (61.05 ± 0.37%), followed by oleic acid (22.99 ± 0.80%), palmitic acid (10.23 ± 0.50%), and stearic acid (5.74 ± 0.23%). There was a statistical significance difference between the fatty acids in the Obeidi seed oil among each other (*p* < 0.05). All the results are in range with the literature, where linoleic acid is reported between 58–78%, oleic acid between 15–20%, palmitic acid between 7–10%, and stearic acid between 4–6%. Figure 2d shows the concentrations of fatty acids derived from the Asswad Karech variety as having the same fatty acids which were shown for the Obeidi variety, but at different concentrations. Linoleic acid was the most abundant (65.16 ± 1.04%) and, in decreasing order, oleic acid (18.99 ± 1.08%), palmitic acid (9.18 ± 0.24%), and stearic acid (6.66 ± 0.20%). In agreement with the Obeidi variety, the fatty acid concentrations in Asswad Karech are in accordance with other grape varieties [19]. A significant difference was also found among the identified fatty acids (*p* < 0.05) for this red variety. When comparing the fatty acid concentrations of both oils, only for stearic acid was no significant difference observed (*p* > 0.05).

As previously reported, grape pomace seeds are a very promising and low-cost source of fatty acids, and their sustainable extraction can have many potential applications in food and nutraceuticals [20]. The high content of unsaturated fatty acids makes it a high-grade nutritional oil because of their beneficial effects on diseases like thrombosis prophylaxis, cardiovascular illness, and high cholesterol in the blood circulation. For example, linoleic acid, the major fatty acid contained in grape seed oil, promotes cardiovascular health by reducing the total cholesterol and low-density lipoproteins (LDL) in animal models [21]. Additionally, plant oils with a high content of polyunsaturated fatty acids, phytosterols, and squalene are usually applied in pharmaceutical and cosmetic industries because consumers nowadays prefer natural products to avoid allergic responses and skin irritations which can be caused by synthetic components [22].

In this work, the lipid extraction was performed with cyclohexane to quantify and identify the amount of lipids. However, for cosmetic and food applications, it is safer and more ecological to use innovative techniques such as supercritical fluid extraction for the recovery of lipids from grape seeds. The lipid extraction was the first step in the multi-step biomass fractionation. The remaining defatted grape seed residue was 85.9 ± 1% (*w*/*w*) of the starting material and was used in the dephenolization step.

#### 2.2.2. Second Step: Polyphenol Extraction from Defatted Grape Seeds

**Figure 3:**
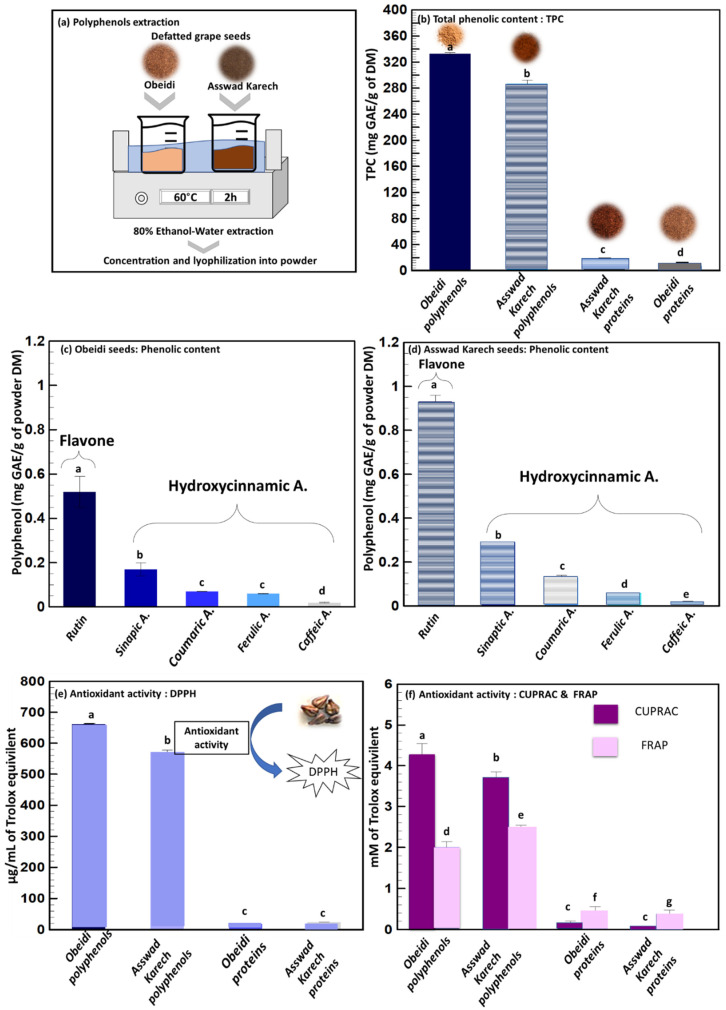
Polyphenol’s extraction. (**a**) Solid–liquid extraction, (**b**) total phenolic content of polyphenol and protein fractions, (**c**,**d**) identification and quantification of phenolic compounds from Obeidi and Asswad Karech polyphenol’s fractions, respectively, and antioxidant activity by (**e**) DPPH assay and (**f**) FRAP and CUPRAC assays of polyphenol and protein fractions. Means sharing the same letter are not significantly different from each other (with *p* > 0.05).

Previous studies concluded that the extraction and recovery of phenolic compounds from defatted seeds is more efficient compared to the recovery from untreated seeds [23]. For this reason, the solid–liquid extraction of the Obeidi and Asswad Karech defatted grape seeds was done as shown in Figure 3a. After the extraction, the concentrates were freeze-dried, and their total phenolic content (TPC) was subsequently determined (Figure 3b). At the end, 44 ± 1 g of polyphenol powder from Obeidi and 24 ± 1 g from Asswad Karech were recovered, and the TPC content of the extract from Obeidi was higher (333.1 ± 1.66 mg GAE/g DM) than for Asswad Karech (287.15 ± 5.18 mg GAE/g DM, *p <* 0.05). According to the literature, these values are in the same range as those reported by Samavardhana et al., who found 362.02 ± 0.79 mg GAE/g DM of defatted (via Soxhlet) red seeds (the Black Queen) extracted with 60% (*v*/*v*) ethanol [23].

In the literature, it is reported that the polyphenol content of grape seeds can be different, and a conclusion cannot be made as to whether the content is higher in red or white varieties. On one hand, some references reported that the seeds of a white grape, Asyrtiko, had a higher TPC compared to the red varieties [24]. On the other hand, other studies reported that the seeds of red grapes, e.g., of Cabernet Franc, had a higher TPC than the seeds from white grape of Vidal Blanc [25].

Figure 3c,d show the identification and quantification of different phenolic compounds by Ultra Performance Liquid Chromatography (UPLC) for the Obeidi and Asswad Karech varieties. Within both grape seeds, the same polyphenols have been identified and they include rutin, being the most abundant, followed in descending order by sinapic acid, coumaric acid, ferulic acid, and caffeic acid. Rutin was present in both extracts, being 0.516 ± 0.07 mg/g DM for Obeidi and 0.925 ± 0.03 mg/g DM for Asswad Karech; the latter was 1.79 times richer in rutin than Obeidi *(p <* 0.05). These results were found to be lower than the one reported by Ozcan et al.: in the Red Globe seeds, a rutin concentration of 2.03 mg/g DM was found after an extraction with methanol, water, and formic acid [26]. However, Rockenbach et al. found a lower concentration of rutin (0.091 mg/g DM) in the red seeds of Cabernet Sauvignon which had been extracted with a mixture of methanol, water, and acetic acid [27]. Rutin is a flavanol widely distributed in various fruits and vegetables, but it is most prevalent in grapes and buckwheat. It is a phenolic compound that possesses a range of beneficial properties, including antioxidant and anti-inflammatory activities. Additionally, rutin demonstrates the ability to protect against cardiovascular diseases, skin cancer, and neurodegenerative disorders [28].

Sinapic acid was the second most abundant phenol observed in both seed extracts: Obeidi contained 0.169 ± 0.03 mg/g DM, whereas Asswad Karech contained 0.291 ± 0.01 mg/g DM. Asswad Karech was 1.72 times richer in sinapic acid than Obeidi *(p <* 0.05). Sinapic acid is a phytochemical which is present in spices, citrus and berry fruits, vegetables, cereals, and oilseed crops that have antioxidant, anti-inflammatory, anticancer, antimutagenic, anti-glycemic, neuroprotective, and antibacterial properties. It can also reduce a variety of chemically induced toxicities [29].

Coumaric acid is a phenolic compound abundantly found in grapes which has preventive effects against chronic diseases and cancer [30]. Coumaric acid was 1.88 times more abundant in the extract from Asswad Karech (0.126 ± 0.001 mg/g DM) than from Obeidi (0.067 ± 0.002 mg/g DM *p <* 0.05). These results are in accordance with the ones found by Ozcan et al. [26] for the Red Globe seeds, with a concentration of 0.0947 mg/g DM and phenolic compounds were extracted using methanol, water, and formic acid. However, Di Stefano et al. found a higher concentration of coumaric acid (4.641 mg/g DM) in the acetone extract of the red defatted Sicilian grape seeds [31].

The amount of ferulic and caffeic acids showed similar concentrations between the extracts from both autochthonous varieties with 0.057 ± 0.004 mg/g DM for Obeidi and 0.062 ± 0.003 mg/g DM for Asswad Karech for ferulic acid *(p >* 0.05), and 0.019 ± 0.001 mg/g DM for Obeidi and 0.023 ± 0.003 mg/g DM for Asswad Karech for caffeic acid *(p >* 0.05). These results were found to be lower than the ones found by Ozcan et al. [26] in the Cinarki Karasi red seeds, with a concentration of 0.45 and 0.59 mg/g for caffeic acid and ferulic acid, respectively. The phenolic compounds from Cinarki Karasi were extracted using methanol, water, and formic acid. Ferulic acid is a non-toxic phenolic compound having a wide range of physiological effects such as anti-inflammatory, antioxidant, antimicrobial, anticancer, and antidiabetic properties. Ferulic acid is a free radical scavenger, as well as an inhibitor of free radical-generating enzymes, and it protects the major skin tissues, including keratinocytes, fibroblasts, collagen, and elastin. It is used as a photoprotective agent, a skin anti-aging, and a brightening component in skin care products. Its application is limited due to its proclivity for oxidation [32]. Caffeic acid, on the other hand, is a phenolic compound produced by all plant species and found in foods like coffee, wine, and tea as it is used as common medications, like propolis. The antioxidant, anti-inflammatory, and anticarcinogenic properties of caffeic acid are well known. Studies have shown that this phenolic compound has an anticarcinogenic effect against a common kind of cancer called hepatocarcinoma (HCC), which is common, aggressive, and causes significant mortality around the world [33].

The divergent results, compared with and mentioned in the literature, are due to the different grape seed varieties and extraction parameters such as the particle size, solid to liquid ratio, time, temperature, and solvents, that are likely to affect the recovery of individual phenolic compounds.

Next to identifying and quantifying the extracted polyphenols, it is important to measure if their biological activity is maintained in order to use them in cosmetic and health applications. The radical scavenging capacity of Obeidi and Asswad Karech extracts were assessed using three different assays (DPPH, CUPRAC, and FRAP) in order to validate the results (Figure 3e,f). The Obeidi extract, the white variety, showed the following antioxidant activity for the three methods used; 662.17 ± 0.01 µg/mL of Trolox equivalent with DPPH, 4.27 ± 0.27 mM of Trolox equivalent with CUPRAC, and 2.01 ± 0.13 mM of iron (II) equivalent with FRAP. The antioxidant activity of the extract from Asswad Karech, the red variety, was 572.26 ± 6.06 µg/mL of Trolox equivalent with DPPH, 3.65 ± 0.20 mM of Trolox equivalent with CUPRAC, and 2.49 ± 0.05 mM of iron (II) equivalent with FRAP. In agreement with the literature, Igua et al. also observed that the antioxidant activity (DPPH) and TPC content of the extract obtained from Romanian white grape seeds, using 80% (*v*/*v*) methanol, were more prominent than that of the extract from the red Romanian varieties [34].

The results show that phenolic rich extract from the autochthonous grape seeds had a high antioxidant activity, which was related to the presence of different phenolic compounds according to the UPLC results. Polyphenols can exsert many benefits on human health, serving as antioxidants, anti-inflammatory, and anti-aging agents, which can be used in food, pharmaceutical, and cosmetic sectors [35,36].

#### 2.2.3. Third Step: Protein Recovery from Defatted and Dephenolized Grape Seeds

**Figure 4:**
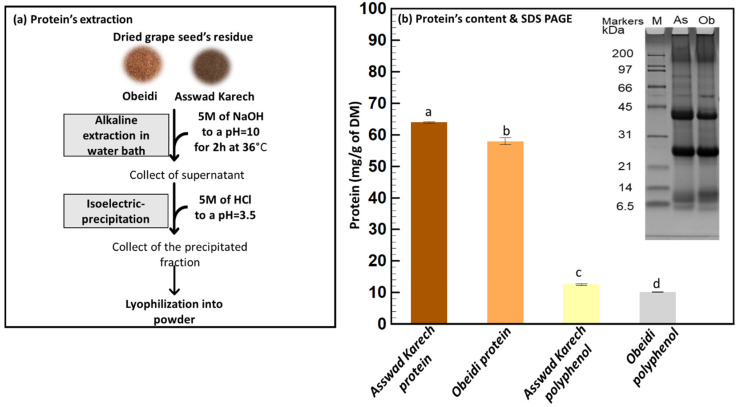
Protein’s extraction. (**a**) Alkaline extraction and isoelectric precipitation and (**b**) quantification and identification using Kjeldhal method and SDS-PAGE, respectively. Means sharing the same letter are not significantly different from each other (with *p >* 0.05).

A previous study confirmed that the protein recovery was more efficient after defatting and dephenolizing grape seeds [37]. For this reason, a protein extraction of the dried residues was taken as a third step following an optimized method (Figure 4b) [37]. This fraction resulted in 3.90% (*w*/*w*) and 4.11% (*w*/*w*) DM freeze-dried powder for Obeidi and Asswad Karech, respectively. It is worth mentioning that even if the relatively high temperatures (60 °C) used in this process might alter the functional properties of the proteins, their nutritional properties are likely to remain [38].

Using the Kjeldhal method, the protein content of the Asswad Karech extract was the highest, 63.95 ± 0.24 mg/g DM, whereas for Obeidi it was 57.93 ± 1.11 mg/g DM, with a significant difference between the two values *(p <* 0.05). SDS-PAGE was performed under reduced conditions to compare the protein profiles of both samples (Figure 4b). They had the same pattern, with only minor differences in the intensities of some bands (e.g., ~10 kDa and 40 kDa). The main bands at ~40 kDa and ~25 kDa correspond with the sub-units of the 11S globulins storage proteins (~65 kDa) from *Vitis vinifera* [39,40]. Although the components of ~40 kDa can also be identified as belonging to the 7S globulin storage proteins, both 7S and 11S globulins have good gelling and emulsifying properties [41].

The grape seed protein fractions include high quantities of essential peptides and amino acids, although the most prevalent amino acids found in grape seeds are glycine, glutamic acid, and aspartic acid [42], and they can be used in the food industries to produce protein supplements after their extraction due to their high nutritional value. Additionally, these compounds can be used as clarifying agents for wine since their effect regulates quality characteristics such as the appearance, color, and stability of red wines [13].

In order to check the quality of the obtained polyphenol and protein powder extracts and the purity of each fraction, the proteins were also measured in the polyphenol powder, and the polyphenols were measured in the protein powder.

The TPC of the protein fraction from Obeidi was 4.43 ± 0.01 mg/g DM and for Asswad Karech 7.43 ± 1.02 mg/g DM, with a significant difference (*p <* 0.05) (Figure 3b). This was also confirmed by the antioxidant activity measured for the protein fractions. Obeidi protein powder had 18.55 ± 2.02 µg/mL of Trolox equivalent using DPPH, 0.17 ± 0.04 mM of Trolox equivalent using CUPRAC, and 0.45 ± 0.11 mM of iron (II) equivalent using FRAP. The protein powder from Asswad Karech had 24.26 ± 0.01 µg/mL of Trolox equivalent using DPPH, 0.09 ± 0.01 mM of Trolox equivalent using CUPRAC, and 0.36 ± 0.11 mM of iron (II) equivalent using FRAP (Figure 3e,f). In both protein factions, a relatively low antioxidative activity was present due to the low number of phenolic compounds which were present. It can be calculated that the protein fractions are 93.0 and 89.6% (Equation (4)) pure in protein from the Obeidi and Asswad Karech seeds, respectively (Table 2).

The protein content of the polyphenol powders was also measured (Figure 4b). The Asswad Karech extract had a protein content of 12.56 ± 0.22 mg/g and the extract of Obeidi had a content of 10.14 ± 0.11 mg/g. There was a statistical significance between the two values (*p* < 0.05) (Figure 4b). The polyphenol powders from Obeidi and Asswad Karech are calculated to be 97.0 and 95.8% (Equation (2)) rich in polyphenols, respectively (Table 2).

The overall multi-step biomass fractionation process allowed for the recovery of a polyphenol powder with almost a 96% purity and a protein powder with a 91% purity (Table 2).

#### 2.2.4. Grape Seed Residue

**Figure 5:**
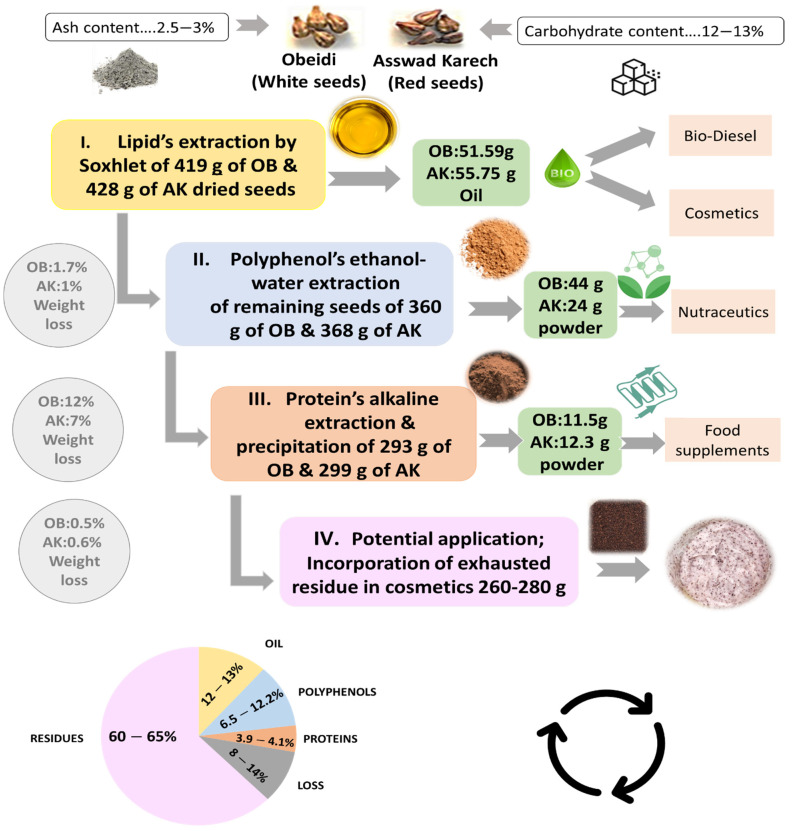
Schematic overview of a multi-step valorization of grape pomace seeds from Asswad Karech (AK) and Obeidi (OB).

Following the lipid, polyphenol, and protein extraction from grape seeds, the exhausted residue was kept and can be, as a potential application, incorporated in a cosmetic product (Figure 5). The carbohydrates content was also performed on the final residues, showing no significant difference with the initial one (Figure 1). After all the extraction steps, most of the carbohydrates remained in the Obeidi and Asswad Karech residues.

## 3. Materials and Methods

### 3.1. Plant Material

Grape pomaces of the Lebanese autochthonous varieties Obeidi (white), and Asswad Karech (red) were provided by Château Saint-Thomas and Chateau Kefraya (Beqaa Valley, Lebanon), respectively, in 2021. All the pomaces were washed and dehydrated at 50 °C for 48 h in an airflow oven. The seeds were separated from the skins using a vibrating multi sieve separator (ELE International, Loveland, CO, USA). The dried seeds were ground using a centrifugal grinding mill ZM 1000 (Retsch^®^ GmbH, Haan, Germany) with a ring sieve of 2 mm. The ground seeds were stored at an ambient temperature in the dark until further use.

### 3.2. Multi-Step Biomass Fractionation Grape Seeds

#### 3.2.1. First Step: Lipid Extraction

A Soxhlet extraction was performed by weighing 500 g of grinded grape seeds of each variety into Whatman Thimbles. The oil/lipids were obtained by a continuous extraction with cyclohexane at 60 °C for 6 h [43]. The solvent was removed with a Rotavapor^®^ R-100 (BUCHI, Swiss) evaporator at 45 °C. The recovered oil was kept at 4 °C until further analysis and the defatted residues were dried at 40 °C. The extracted quantity of the lipids was expressed in a mass percentage as follows:

(1) $$\mathrm{Lipids}\%\;\left(\frac{w}{w}\right)=\frac{\mathrm{Mass}\;\mathrm{of}\;\mathrm{oil}}{\mathrm{Mass}\;\mathrm{of}\;\mathrm{seeds}\;\mathrm{in}\;\mathrm{Dry}\;\mathrm{Matter}}\times 100\%$$

#### 3.2.2. Second Step: Polyphenol Extraction from Defatted Grape Seeds

Conventional solid–liquid extraction was done using the remaining defatted residues in 80% (*v*/*v*) ethanol [44]. The solid-to-liquid ratio was 1:5 (*w*/*v*). The polyphenols from the seeds of both varieties were extracted at 60 °C for 2 h. The obtained extract was centrifuged at 15,000× *g* for 10 min at room temperature. The supernatant was collected, and the ethanol was removed by a vacuum Rotavapor^®^ R-100 (BUCHI, Swiss) at 40 °C. The water extracts were freeze-dried, and the recuperated powder was stored in the dark at room temperature for further analysis. The remaining residues obtained were dried at 40 °C for the protein extraction.

#### 3.2.3. Third Step: Extraction of Proteins from Defatted and Dephenolized Grape Seeds

The dried residues, obtained after the polyphenol extraction, were subsequently used for the extraction of the proteins. The extraction was accomplished by soaking the residues in alkaline water, followed by isoelectric precipitation. The proteins were extracted with Milli-Q water with a 1:9 (*w*/*v*) ratio at 36 °C for 2 h after an adjustment of the mixture to a pH value of 10 with sodium hydroxide (5 M). The pH was kept constant during the extraction procedure. Hereafter, the samples were centrifuged at 17,500× *g* at 4 °C for 20 min and the supernatants were collected. The pH of the supernatants was adjusted to pH 3.5 with hydrochloric acid (5 M), and at this pH, the proteins precipitated and were collected after centrifugation at 17,500× *g* at 4 °C for 20 min [37]. The collected proteins were dissolved in Milli-Q and the pH was adjusted to a pH of 7, and the extract was freeze dried into a powder. The protein extracts were stored in the dark at room temperature before further analysis.

#### 3.2.4. Exhausted Grape Seed Residue

The final exhausted grape seed residues were washed with Milli-Q water and adjusted to a pH of 7 and subsequently dried at 60 °C. The residues were tested for applications in cosmetics, to replace the microbeads in scrub formulations.

### 3.3. Dry Matter and Ash Content

Five grams of each sample was weighed and left for 24 h in a drying oven (Nabertherm^®^ GmbH, Haan, Germany) at 105 °C. Hereafter, the samples were cooled down in a desiccator until they were at room temperature (RT) and the weight of the samples was determined.

Ash was determined by incineration in a muffle furnace at 525 °C for 4 h, followed by another 4 h at 900 °C. The residues were cooled in a desiccator for 24 h and weighed.

### 3.4. Carbohydrate Identification and Quantification

About 80–100 mg of ground grape seeds (initial raw material) of both varieties, Obeidi and Asswad Karech, were mixed with 5 mL 72% (*w*/*w*) sulfuric acid and incubated at 35 °C for 1 h. Hereafter, the sulfuric acid was diluted until they were at 2 M by adding 25 mL of Milli-Q water, and the mixtures were incubated for another hour at 100 °C. The samples were cooled on ice after hydrolysis and centrifuged (3000× *g*, 15 min, room temperature) (Figure 1a). The supernatant of each sample was used for the analysis of the sugar composition. Before the analysis, all samples were diluted by adding 150 µL of sample to 4.85 mL of Milli-Q water, after that, 5 µL of 0.5% (*w*/*v*) bromophenol blue in 20% (*v*/*v*) ethanol was added. To adjust the pH, solid barium carbonate was added until a clear blue color was obtained. The samples were filtered using an 0.45 µm PTFE filter for High Performance Anion Exchange Chromatography (HPAEC) analysis [45].

The carbohydrate composition was determined according to Keijzer et al. [46]. An HPAEC system equipped with a CarboPac PA-1 column (2 × 250 mm) in combination with a CarboPac PA guard column (2 × 25 mm) and a pulsed electrochemical detector in pulsed amperometric detection mode (Dionex, Sunnyvale, USA) was used. The detection of the monomers was possible after the post-column addition of 500 M of sodium hydroxide at a flow rate of 0.1 mL min^−^^1^. The elution flow rate was set at 0.3 mL min^−^^1^. The elution was done using a mobile phase A of 500 mM NaOH, a mobile phase B of 150 mM NaOH, and a mobile phase C of water. The elution was performed by running for 53 min 100% of C, subsequently 100% of B for 10 min, followed by 100% of A for 15 min, and 100% of C for 22 min. Each sample was analyzed in triplicate.

### 3.5. Fatty Acid Identification and Quantification

Ten mg of each grape seed oil was taken and 2 mL of sulfuric acid (15% (*w*/*v)*) in methanol was added to convert the fatty acids into their methyl esters. All samples were incubated and stirred at 85 °C for 4 h and hereafter cooled on ice. Milli-Q water (1 mL) was added to the samples and subsequently centrifuged for 5 min at 1000× *g* at 24 °C. The lower layer was taken for GC analysis. As standards, 100 mg of a fatty acid methyl ester mixture C8-C24 was used (Supelco, Merck, Darmstadt, Germany). Methyl pentadecanoate was used as the internal standard (Sigma Aldrich, Schnelldorf, Germany). The analysis was done using a 1300 GC Thermo^®^ Scientific Trace GC coupled to a flame-ionization detection detector and a 30 m ZB-fatty acid methyl ester column (Zebron, Phenomenex, Utrecht, the Netherlands). An injection volume of 1 µL with a 1:20 split rate was used. Chloroform was used as a solvent and helium was used as a carrier gas with a flow rate of 1.20 mL min^−^^1^. The program used was as follows: 2 min at 100 °C, then the temperature was increased to 140 °C in 4 min, after which the temperature was increased to 190 °C within 16 min. Hereafter, the column was purged at 260 °C for 4 min.

### 3.6. Total Phenolic Content

The TPC was quantified using the Folin–Ciocalteu method [47]. Two hundred µL of the extracts (polyphenol and protein) and 1000 µL of a ten-fold diluted Folin–Ciocalteu reagent (Sigma-Aldrich, Darmstadt, Germany) were added to 800 µL of sodium carbonate solution (75 g L^−^^1^). The mixture was incubated at 60 °C for 10 min and hereafter cooled at 4 °C for 10 min. The absorbance was measured at 750 nm and gallic acid was used as standard. The TPC is expressed as milligrams of gallic acid equivalents (GAE) per gram of dry matter (mg GAE/g DM).

The polyphenol purity of the powder was calculated according to Equation (2) [48]:

(2) $$\mathrm{Polyphenol}\;\mathrm{purity}\%=\frac{\mathrm{Total}\;\mathrm{phenolic}\;\mathrm{content}}{\mathrm{Total}\;\mathrm{phenolic}\;\mathrm{content}+\mathrm{protein}\;\mathrm{content}}\times 100\%$$

### 3.7. Identification and Quantification of Phenolic Compounds

A hundred mg of each phenolic extract was dissolved in 10 mL 50% (*v*/*v*) acetonitrile and filtered using a 0.45 µm syringe filter. The identification and quantification of phenolic compounds was performed using a Thermo^®^ Scientific UPLC (Vanquish, ThermoScientific, Breda, the Netherlands) equipped with a Waters Acquity BEH C18 column (150 × 2.1 mm, 1.7 µm; Waters, France). The mobile phase A contained 0.1% (*v*/*v*) formic acid and phase B 100% acetonitrile, an injection volume of 10 μL, and a constant flow rate of 0.35 mL min^−^^1^ was used. The gradient (*v*/*v*) was obtained, increasing phase B from 4% in 2 min; to 60% in 28 min; to 100% in 5 min; and back to 4% in 5 min. The absorbance was measured at 280 nm due to a strong interference at 210 nm. All samples were analyzed in triplicate.

### 3.8. Protein Identification and Quantification

The protein content was determined using the protein and polyphenol extracts collected from both varieties by the Kjeldahl method. The protein content was obtained using the standard nitrogen-to-protein conversion factor of N × 6.25, according to Equation (3) [49]:

(3) $$\mathrm{Protein}\;\mathrm{content}\;(\mathrm{mg}\;g{-}^{1})=\frac{\left({\mathrm{mL}}_{\mathrm{HCl}}-{\mathrm{mL}}_{\mathrm{blank}}\right)\times 0.1\times 14\times 6.25}{m_{\mathrm{powder}}\times \mathrm{DM}\%}$$

The protein purity of the powder was calculated according to Equation (4) [48]:

(4) $$\mathrm{Protein}\;\mathrm{purity}\%=\frac{\mathrm{Protein}\;\mathrm{content}}{\mathrm{Total}\;\mathrm{phenolic}\;\mathrm{content}+\mathrm{protein}\;\mathrm{content}}\times 100\%$$

### 3.9. Biological Activities of Polyphenol and Protein Extracts

#### 3.9.1. Diphenyl-2-Picrylhydrazyl Free Radical Scavenging Activity (DPPH)

The ability of the phenolic compounds to decrease DPPH (2,2-diphenyl-picrylhydrazyl) was used to determine their free radical scavenging activity. The DPPH solution (1450 μL, 0.06 mM) was added to 50 μL of solution of grape seed extract or Trolox (positive control, Sigma-Aldrich, St-Quentin Fallavier, France) [50]. The absorbance was measured at 515 nm after 30 min of incubation at room temperature in the dark. A calibration curve was obtained using different concentrations of Trolox. The DPPH free radical inhibition was calculated according to Equation (5):

(5) $$\mathrm{Inhibition}\;\mathrm{percentage}\;\left(\%\right)=\frac{\left(\mathrm{absorbance}\;\mathrm{of}\;\mathrm{negative}\;\mathrm{control}-\mathrm{absorbance}\;\mathrm{of}\;\mathrm{sample}\right)}{\mathrm{absorbance}\;\mathrm{of}\;\mathrm{negative}\;\mathrm{control}}\times 100\%$$

#### 3.9.2. Ferric Reducing Antioxidant Power Assay (FRAP)

The antioxidant activity of the compounds, that can reduce the ferric complex at an acidic pH in the presence of a suitable antioxidant solution, was measured using the FRAP antioxidant capacity kit (Bioquochem, Asturias, Spain). The reaction is fast and proportional to the antioxidant capacity of the samples. In a nutshell, 10 μL of diluted extract (or standard) was mixed with 220 μL of ready-to-use FRAP working solution. After 4 min of continuous stirring, the absorbance was measured at 593 nm. A calibration standard curve was made using the association between the absorbance and the iron II concentrations (Bioquochem, Asturias, Spain). The iron (II) equivalents (mM iron (II)) were the units which was used to measure the antioxidant activity.

#### 3.9.3. CUPRIC Ion Reducing Antioxidant Capacity Assay (CUPRAC)

The antioxidant activity of the extracts was further measured using a CUPRAC assay kit (Bioquochem, Asturias, Spain). CUPRAC uses the oxidation of copper (II)-neocuproine (2,9-dimethyl-1,10-phenanthroline) to determine the total antioxidant capacity. To 200 μL of working solution, 40 μL of the diluted extract, or standard, was added. After 30 min of incubation at room temperature, the absorbance was measured at 450 nm. The results were given in milligrams of the Trolox equivalents (mM TE).

### 3.10. Statistical Analyses

STATGRAPHICS^®^ Centurion XVI was used for variance analysis (one-way ANOVA) and the Least Significant Difference test (StatPoint Technologies, Inc.). The averages of the bar graphs that share the same letter are not significantly different from each other (*p* > 0.05).

## 4. Conclusions

The multi-step biomass fractionation of the seeds from the pomace of two autochthonous grape varieties, Obeidi (white) and Asswad Karech (red), was carried out successfully (Figure 5). Three different fractions consisting of grape seed oil, polyphenol extract, and protein extract were recovered from these wine-making wastes. The amount of oil recovered was 12.3% (*w*/*w*) from Obeidi and 13.0% (*w*/*w*) from Asswad Karech. For the Obeidi seeds, the yield of polyphenol extract was 12.0% (*w*/*w*) and that of the protein extract was 3.9% (*w*/*w*). For the Asswad Karech seeds, the yield of the polyphenol extract was 6.6% (*w*/*w*) and that of the protein extract was 4.1% (*w*/*w*). Moreover, this study revealed that > 90% enriched polyphenol and protein extracts were obtained, with a total extraction loss of 14.2% (*w*/*w*) for Obeidi and 8.5% (*w*/*w*) for Asswad Karech. These results prove that despite the great disposal of waste by the wine industry, it is still possible to recover a promising number of bioactive fractions which have a potential application in the cosmetic, food, and nutraceutical markets. Furthermore, the remaining residue can be valorized to be added to a cosmetic formulation as a replacement for plastic microbeads. In conclusion, the designed multi-step biomass fractionation has the potential to improve the valorization of the side products (grape seeds) of these two Lebanese autochthonous grape varieties.

## Figures and Tables

**Table 1:** Dry matter and ash content (% *w*/*w*) of grape seeds from pomace of Obeidi and Asswad Karech.

	Obeidi	Asswad Karech
Dry matter content	88.57 ± 0.45	88.32 ± 0.46
Ash content (525 °C)	3.00 ± 0.11	2.96 ± 0.06
Ash content (900 °C)	2.29 ± 0.11	2.65 ± 0.49

**Table 2:** Amount and purity of polyphenol and protein fractions from Obeidi and Asswad Karech seeds.

	Polyphenol Fraction		Protein Fraction	
	Obeidi	Asswad Karech	Obeidi	Asswad Karech
Weigh of fraction (g)	44.0 ± 1.0	24.0 ± 1.0	11.5 ± 1.0	12.3 ± 1.0
Total phenolic content (mg/g extract DM)	333.1 ± 1.66	287.15 ± 5.18	4.43± 0.01	7.43 ± 1.02
Polyphenol purity (Equation (2))	97.0%	95.8%	7.0%	10.4%
Protein content (mg/g extract DM)	10.14± 0.11	12.56 ± 0.22	57.93 ± 1.11	63.95 ± 0.24
Protein purity (Equation (4))	3.0%	4.2%	93.0%	89.6%

## Data Availability

Not applicable.
